# Managing low corneal astigmatism in patients with presbyopia correcting intraocular lenses: a narrative review

**DOI:** 10.1186/s12886-023-03003-2

**Published:** 2023-06-06

**Authors:** Carlos Rocha-de-Lossada, Manuel Rodríguez-Vallejo, Marina Rodríguez-Calvo-de-Mora, Filomena J Ribeiro, Joaquín Fernández

**Affiliations:** 1 Qvision, Department of Ophthalmology of VITHAS Almería Hospital, Almería, 04120 Spain; 2 Ophthalmology Department, VITHAS Málaga, Málaga, 29016 Spain; 3 Hospital Regional Universitario de Málaga, Plaza del Hospital Civil, S/N, Málaga, 29009 Spain; 4 Departamento de Cirugía, Universidad de Sevilla, Área de Oftalmología. Doctor Fedriani, S/N, Sevilla, 41009 Spain; 5 Departamento de Oftalmologia, Hospital da Luz, Lisbon, 1500-650 Portugal; 6 Faculdade de Medicina, Universidade de Lisboa, Lisbon, 1649-028 Portugal

**Keywords:** Astigmatism, Presbyopia, Multifocal, Intraocular lenses, Cataract surgery

## Abstract

Cataract surgery has become a refractive procedure in which emmetropia is the goal, with the implantation of extended depth-of-focus or multifocal intraocular lenses (IOLs) being the commonly selected option to restore vision beyond the far distance. The selection criteria for implanting these lenses can differ from those for monofocal IOLs and even between technologies, as eye characteristics can affect postoperative visual performance. Corneal astigmatism is an eye characteristic that can affect visual performance differently, depending on the implanted IOL. The magnitude of corneal astigmatism, the tolerance of the IOL to this astigmatism, economic aspects, comorbidities, and the efficacy of astigmatism treatment are factors that can make surgeons’ doubt as to what astigmatism treatment should be applied to each patient. This review aims to summarize the current evidence related to low astigmatism tolerance in presbyopia-correcting lenses, the efficacy achieved through corneal incisions, and their comparison with the implantation of toric IOLs.

**Supplementary Information:**

The online version contains supplementary material available at 10.1186/s12886-023-03003-2.

## Introduction

Cataract surgery, which is the most commonly performed surgery worldwide, has become a refractive procedure with emmetropia as the goal [1]. A monofocal intraocular lens (IOL) is often selected to replace the cataractous crystalline lens to restore distance vision; however, the patient still requires spectacle correction to obtain clear vision at intermediate and near distances. Both extended depth-of-focus (EDOF) and multifocal intraocular lenses (MIOLs) allow the patient to restore vision from far to intermediate and/or near distances, respectively. Therefore, implantation of a MIOLs or EDOF IOLs is an alternative treatment for cataracts and presbyopia correction, even with a clear crystalline lens.

Corneal astigmatism can affect the visual performance of patients depending on the implanted IOL [2, 3]. According to the EUREQUO database, more than 30% of pseudophakic patients may have a residual astigmatism greater than 1.0 diopter [4]. The most effective approach to correct this astigmatism is the implantation of a toric IOL (TIOL) [5]. Regarding the correction of a low amount of astigmatism (< 1.5 D) with TIOLs, between 72.3% and 84% of the patients can achieve a postoperative refractive cylinder lower than 0.5 D [6–9]. This achievement represents between 14% and 22.8% more than the use of a spherical IOL (70% and 49.5%) [6, 7, 9]. Higher percentages of accuracy have been reported for Restor SND1T2 MIOL in comparison to a monofocal IOL, 94.7% and 88.7%, respectively, but the sample of eyes was below 1.18 D of corneal astigmatism with a mean around 0.47 D for the MIOL group [10]. Kalaydzhiev et al. also reported accuracy of 100% but for inclusion criteria below 0.75 D of corneal astigmatism [11]. However, the use of TIOL might be questionable in some circumstances, such as pseudoexfoliation, zonulophathy or a small pupil among others [12]. In addition, the increase in the cost of the procedure might lead the surgeon to achieve this correction through corneal incisions that flatten the steepest meridian of the cornea,[13, 14] especially in common cases of low levels of corneal astigmatism, between 0.50 D and 1.50 D, presented in two-thirds of eyes submitted to cataract surgery [15]. For this issue, the management of corneal astigmatism can be achieved through manual and/or femtosecond laser (FSL) incisions [16–18].

Anterior segment surgeons might have doubts about which treatment approach to follow, especially in low levels of corneal astigmatism,[5, 12] considering the tolerance of each presbyopia correcting IOL and the efficacy of corneal incisions versus implanting a toric IOL. The purpose of this review is to explain how levels of low corneal astigmatism (< 1.50 diopters) can affect the visual performance achieved with different IOL technologies, to describe the effectiveness of different types of corneal incisions to reduce the final refractive or corneal astigmatism, and to compare this with the alternative implantation of a toric IOL.

## Methods

The following questions were addressed in this narrative review:

- What is the effect of astigmatism magnitude on the visual performance, depending on the implanted intraocular lens?
- What is the accuracy of astigmatism correction achieved through the application of corneal incisions in low corneal astigmatism?
- How comparable is the accuracy of corneal incisions compared to the correction with TIOL?

Owing to the wide scope of this narrative review, two separate searches were conducted by two different reviewers. The search strategy, inclusion criteria, and data extraction can be found in the supplemental material.

## Tolerance to astigmatism

Refractive astigmatism correction above 0.5D improves optical quality and therefore the visual acuity, in comparison to the spherical equivalent correction in phakic young patients [19, 20]. The decrease in optical quality and visual acuity can be similar regardless of the astigmatism type and optotype distance for moderate astigmatism (2.00 D),[21] but the reading performance has been reported to be higher in the case of simple myopic against the rule astigmatism (ATR) in this case [21]. These results are in agreement with those reported by Singh et al. [22] who found that an astigmatism above 1.00 D decreased uncorrected distance visual acuity (UDVA) without a benefit for uncorrected near visual acuity (UNVA). With regard to low astigmatism, UDVA has also shown a higher tolerance for with the rule (WTR) astigmatism (0.75 D) in comparison to ATR (0.50 D) astigmatism [20]. These differences on UVAs between astigmatism types and distances can be explained by the optotypes or reading charts used. An eye with simple myopic WTR residual astigmatism has the vertically extended point spread function (PSF) closer to the retina, and therefore a lower crowding effect in a far distance ETDRS chart with letters less separated on the horizontal than on the vertical [21]. Conversely, with the same WTR astigmatism, the PSF extended horizontally will be closer to the retina with the patient reading at near vision and therefore a higher crowding effect at near vision [21]. Although these studies were based on simulated astigmatism, low residual ATR astigmatism (< 1.25 D) has also been reported to benefit near vision in pseudophakic patients implanted with monofocal IOLs in combination with a myopic shift between 0 and − 0.50 D [23, 24]. In conclusion, targeting to a myopic simple (-1.50@90º) or mixed ATR astigmatism around 1 D with myopic spherical target <|-0.50| D could be considered an option for increasing depth of focus with monofocal IOLs (i.e. -0.25, -1.00@90º) [25].

### Presbyopia correcting intraocular lenses

Previous recommendation for targeting to astigmatism residual to extend depth-of-focus with monofocal IOLs is not transferable to multifocal IOLs [26, 27]. Although Berdahl et al., in a large-scale data study based on uncontrolled entries on a website, reported that the tolerance of UDVA to astigmatism was similar between MIOLs and monofocal IOLs,[28] this finding is inconsistent with those reported in other large-scale data and simulation studies [29, 30]. Schallhorn et al. reported, in a study with a large sample size implanted with multiple presbyopia correcting IOLs, a slightly higher tolerance of residual astigmatism with MIOLs than with monofocal IOLs, regardless of the type of astigmatism [30]. However, the percentage of patients who were satisfied or very satisfied only decreased by 6% when the residual astigmatism was ≥ 0.75 D, even though the percentage of eyes achieving 20/20 vision decreased by 19.5%.

It is noteworthy that Schallhorn et al. study included a sample with the majority of IOLs being EDOF or low-addition bifocal IOLs, and Muftuoglu et al. reported a higher tolerance to simulated ATR astigmatism over UDVA as the IOL addition decreased, and conversely at UNVA even though differences were smaller in the latter case [31]. These findings align with those reported by Carones et al., who found a higher tolerance to simulated astigmatism in the following order: Symfony, ReSTOR 2.5, ReSTOR 3.0 and PanOptix,[32] as well as the Zemax simulations comparing EDOF and ReSTOR 3.0 [33]. The induction of 0.75 D reduced one line of UDVA in the Symfony, whereas the same decrease was obtained for 0.50 D in the case of Restor and PanOptix [32]. This lower tolerance to astigmatism might lead to decreased satisfaction in the presence of astigmatism with bifocal lenses, as was reported by Carones et at. and confirmed by Xu et al. [32, 34]. However, McNeely et al. also reported a high tolerance to residual astigmatism for the bifocal Mplus LS-312 MF30 and Pedrotti et al. for the Precizon, comparable to that reported for Symfony,[27, 32, 35] thus the tolerance extends beyond the addition and might involve the optical design. In this regard, the highest tolerance to residual astigmatism has been reported for the small-aperture IC-8, which saw a decrease of one line of UDVA for an astigmatism of 1.5 D [36].

Although the decrease of addition might suggest a higher tolerance to astigmatism,[31, 32]. Hayashi et al. [37] have conversely reported in a simulation study, a lower tolerance of UDVA for ReSTOR + 3 than + 4. This indicates that the higher tolerance of UDVA to astigmatism with the decrease of addition may at least be considered as controversial. On the other hand, the lower tolerance to astigmatism on UDVA for PanOptix in comparison to Restor + 3 previously described was also confirmed in a simulation study of Hayashi et al. [32, 38]. One of the most important findings in Hayashi et al. studies was that, even though UDVA continuously decreases with astigmatism induction, the near and intermediate ranges were more tolerant to astigmatism induction [37, 38]. Another relevant study describing the influence of astigmatism beyond UDVA was conducted by Xue et al. [39]. The authors reported that a main corneal incision of 2.8 mm over the steep meridian in the implantation of the AT Lisa tri 839MP reduced the corneal astigmatism from 0.73 D to 0.44 D. This led to better uncorrected intermediate visual acuity (UIVA) in comparison to an oblique incision (135º) even though the magnitude of differences was reduced from 1 day (0.1 logMAR) to 3 months (0.05 logMAR).

## Astigmatism correction with corneal incisions

Corneal incisions are based on the coupling effect, which means that an incision flattens the steep meridian while steepening the flatter meridian 90 degrees away from the incision [40–42]. If the incisions induce twice as much flattening as steepening, the coupling ratio is 2:1. Conversely, when the ratio is equal, a 1:1 ratio is produced [42]. Various techniques of corneal incisions have been developed. Among them, the Single Clear Corneal Incision (CCI), Opposite CCI (OCCI), Arcuate Keratotomy (AK) and Limbal Relaxing Incision (LRI) stand out, either as manual or through the use of femtosecond laser surgery (FLS). In this context, a systematic review demonstrated that both manual and FLS AK are safe and moderately effective with similar correction index around 0.7 on the correction of corneal astigmatism during cataract surgery [43]. Unlike this systematic review, our narrative review encompasses a wider scope in the astigmatism treatment types, only including studies focused on low astigmatism. Moreover, in light of the similar correction indexes reported in the previous systematic review,[43] both FLS and manual incisions are detailed together in sections of each incision type.

### Single clear corneal incisions (CCI)

An incision in the clear cornea produces a flattening of the corneal curvature in that meridian [44]. This flattening may be influenced by different factors such as incision size, [45], [46] shape,[44, 46] location relative to the limbus [44] or to the preoperative corneal astigmatism [44], [47]. As illustrated in Table 1, the location of the CCI varies according to the preferences of the surgeon, achieving the highest reduction of astigmatism with the CCI located at the steepest meridian. Regarding the size of the incision, there is great variability between studies, from 1.8 mm [48] to 5.5 mm [49]. However, most of the studies collected use a size between 2.2 and 3.5 mm (Table 1). The arithmetic difference between the preoperative and postoperative corneal astigmatism was below 0.5 D in the 13 retrieved studies, with only three studies (23%) reporting a difference greater than or equal to 0.3 D [50–52]. Furthermore, only one study reported the percentage of subjects achieving postoperative refractive astigmatism in 0.25 D (34%), 0.50 D (56%), 0.75 D (72%), and 1 D (91%). These percentages for CCI of 3 mm at the steepest meridian were below those obtained with TIOL, 0.25 D (47%), 0.50 D (75%), 0.75 D (89%), and 1 D (100%) with temporal CCI of 2.4 mm.

**Table 1:** Preoperative and postoperative magnitude of corneal astigmatism reported in clear corneal incision (CCI) studies

Author	Year	Study Type	Eyes	Follow-up (m)	Size(mm)	Location	Preop. Type	Metric	Device	Pre	Post	Diff
Nielsen[50]	1995	RCT	12	1	3,5	Temporal	ATR	K	Allergan-Humphrey	0,92	0,41	-0,5
Nielsen[50]	1995	RCT	11	1	5,2	Superior	WTR	K	Allergan-Humphrey	0,96	0,47	-0,5
Wei[51]	2012	CS	18	3	2,5	Temporal	ATR	K	TMS-4	1,14	0,74	-0,4
Li[52]	2019	CS	104	3	2,4	Steepest-one-handed	All	TCA	Pentacam	1,22	0,94	-0,3
Li[52]	2019	CS	105	3	2,4	Steepest-two-handed	All	TCA	Pentacam	1,17	0,92	-0,3
Li[53]	2019	CS	68	3	2,4	Steepest	All	K	Pentacam	1,08	0,87	-0,2
Simon[44]	2005	CS	23	7,8	3,2	Steepest	All	K	Videokeratometry or Javal	0,9	0,7	-0,2
Li[53]	2019	CS	68	3	2,4	Steepest	All	TNP	Pentacam	1,21	1,02	-0,2
Piao[54]	2020	CS	49	2	3,2	Steepest	ATR	K	Pentacam	0,76	0,6	-0,2
Piao[54]	2020	CS	61	2	3,2	Steepest	Oblique	K	Pentacam	0,81	0,71	-0,1
Piao[54]	2020	CS	49	2	3,2	Steepest	ATR	TCRP3	Pentacam	0,88	0,79	-0,1
Yoon[55]	2013	CS	30	3	3	Temporal	All	K	ARK-510 A	0,71	0,64	-0,1
Lee[56]	2019	CS	26	1	2,2	NA	All	K	Canon R-50	0,76	0,72	0,0
Ozyol[57]	2012	RCT	24	2	3	Superior	ATR	K	Manuel keratometry	0,99	0,99	0,0
Febbraro[48]	2015	RCT	63	1	1,8	Superior	All	K	TonorefII	0,65	0,67	0,0
Wei[51]	2012	CS	18	3	2,5	Temporal	All	K	TMS-4	0,82	0,84	0,0
Wang[58]	2019	CS	27	3	3	Superotemporal (0,5 limbus)	All	K	Pentacam	0,86	0,92	0,1
Wang[58]	2019	CS	27	3	3	Superotemporal (1–1,5 limbus)	All	K	Pentacam	0,86	0,94	0,1
Kamiya[59]	2021	CS	200	3	2,8	Temporal	All	K	TONOREFF-II	0,66	0,74	0,1
Yang[60]	2017	RCT	30	1	2,2	Superior	All	K	Pentacam	0,93	1,02	0,1
Beltrame[49],	2001	RCT	60	3	3,5	Superonasal	All	K	EyeSys	0,78	0,88	0,1
Piao[54]	2020	CS	61	2	3,2	Steepest	Oblique	TCRP3	Pentacam	0,8	0,91	0,1
Hayashi[61]	2015	CS	102	60	4,1	Temporal	All	K	ARK-700 A	0,87	0,98	0,1
He[62]	2021	RCT	70	3	3,2	Temporal	All	K	NA	0,89	1,04	0,2
Febbraro[48]	2015	RCT	61	1	3,2	Superior	All	K	TonorefII	0,69	0,94	0,3
Ozyol[57]	2012	RCT	24	2	3	Superior	WTR	K	Manuel keratometry	0,83	1,2	0,4
Beltrame[49]	2001	RCT	54	3	5,5	Superonasal	All	K	EyeSys	0,7	1,97	1,3

CS: Case Series; RCT: Randomized clinical trial; K: Keratometry; TCA: Total corneal astigmatism; TNP: Total net power; TCRP3: Total corneal refractive power at 3 mm; ATR: Against-the-rule; WTR: With-the-rule; NA: Not available; Diff: Difference between posteroperative (Post) and preoperative (Pre) magnitude of corneal astigmatism.

### Opposite clear corneal incisions (OCCI)

An enhancement of the CCI technique involves the use of a supplementary incision in the opposite corneal axis, a procedure termed OCCI [63] In regard to OCCI, Mendicute et al. detailed their personalized nomogram, which consists of making a 2.75 mm incision for astigmatism ranging from 1.00 to 1.75 D [64]. A second CCI of identical size was made 180 degrees from the first incision, thereby creating an OCCI. The concept is to adopt a surgical approach based on the orientation of the preoperative astigmatism. In cases with WTR astigmatism, the superior approach is employed, whereas in instances of ATR astigmatism, the temporal incision is selected. Consequently, in cases of oblique astigmatism, the superior temporal incision is the preferred choice [64]. Only a few studies have reported on the accuracy of OCCI in low corneal astigmatism (Table 2), and these were applied to a mean preoperative corneal astigmatism ≥ 0.99 D. A single study reported the percentage of subjects achieving postoperative refractive astigmatism in 0.25 D (9%), 0.50 D (54%), 0.75 D (75%), and 1 D (89%) [65]. To the best of our knowledge, no studies have compared the accuracy of OCCI with the implantation of a TIOL.

**Table 2:** Preoperative and postoperative magnitude of corneal astigmatism reported in opposite clear corneal incision (OCCI) studies

Author	Year	Study Type	Eyes	Follow-up (m)	Size(mm)	Location	Preop Type	Metric	Device	Pre	Post	Diff
Chen[65]	2020	CS	138	1	2,8	Steepest	All	K	Pentacam	1,31	0,69	-0,6
Ren[66]	2019	RCT	28	3	3	Steepest	All	K	Pentacam	1,09	0,52	-0,6
Ren[66]	2019	RCT	40	3	2	Steepest	All	K	Pentacam	0,99	0,69	-0,3
Nemeth[67]	2014	CS	81	2	3	Steepest	All	K	IOL Master	1,06	0,86	-0,2

CS: Case Series; RCT: Randomized clinical trial; K: Keratometry; NA: Not available; Diff: Difference between posteroperative (Post) and preoperative (Pre) magnitude of corneal astigmatism

### Arcuate keratotomy (AK)

The manual AK consists of a non-penetrating corneal incision, around 90–95% depth, with a variable length and an arc shape created with a calibrated diamond knife performed in the steep corneal meridian around 7-9-mm optical zone [40]. This technique has been reported to be a safe, effective, and stable procedure for reducing corneal astigmatism during phacoemulsification. [68], [69]. Some studies have shown that manual AK does not induce higher-order aberrations in the long term, making it a safe and long-lasting treatment [70]. However, manual AK may exhibit low reproducibility, high variability and may be highly surgeon dependent [71]. Likewise, AK may be performed with the use of FLS which has proven to be more reproducible [43].

Several years ago, Maloney et al. reported their results using different AK techniques. In terms of AK, they started with two pairs of 3 mm transverse incisions 180 degrees apart on either side of the visual axis tangent to the 7.0 and 8.0 mm optical zones. However, due to the fact that these patients were overcorrected, they began to perform one pair of transverse incisions tangent to the 7.0 mm optical zone [72].

Years later, Amigo et al. reported their normogram in eyes with ATR astigmatism. For this, in eyes with a magnitude between 0.5 and 1.25 D, an incision with an arc length of 45 degrees was used, while in astigmatisms > 1.25 D the arc length was 55 degrees. [73]. Chen et al., [68] developed with the help of the Optiwave Refractive Analysis (ORA) and digital eye tracking (VERION) a new nomogram based on a previous one created by the same authors that was not published previously in the literature. This new nomogram involves using an incision of 45°±2.5° at 9 mm in cases of astigmatism ATR whose magnitude is between 1.00 and 1.25 diopters, while the arc must be 35°±2.5° when the magnitude is between 0.5 and 0.75 diopters. In the case of WTR astigmatism, two incisions are needed in magnitudes between 1.00 and 1.25 D with an arc length of 15°×2 at 9 mm and one incision when the magnitude is between 0.5 and 0.75 D with an arc length of 25°±2.5° at 9 mm. [68]. Similarly, Kwitko et al. several years ago also reported their own nomogram which used an optical zone of 7 mm to correct corneal astigmatism up to 1.5D [74]. Table 3 shows the accuracy reported by several studies achieving a correction above > 0.5D in around the 60% of the included studies.

**Table 3:** Preoperative and postoperative magnitude of corneal astigmatism from keratometry reported in arcuate incision (AK) studies

Author	Year	Study Type	Eyes	Age_M	Technology	Follow (m)	CCI Size	Location	Nomogram	Device	Pre	Post	Diff
Wendelstein[12]	2021	CS	43	69	Victus	3	2,2	Temporal	Castrop	Lenstar	1,45	0,4	-1,05
Visco[75]	2019	CS	189	68,3	Lensar	3	NA	NA	Paired	Cassini	0,92	0,14	-0,78
Wang[76]	2022	RCT	45	67	Catalys	1	2,4	Temporal	Paired 9 mm	IOL Master	1,04	0,28	-0,76
Chen[68]	2019	CS	60	68	Manual	3	NA	NA	Verion guide	IOLMaster	1,1	0,37	-0,73
Wang[77]	2018	CS	25	68,56	LenSX	3	3	Steeper	Donnenfeld	OPD-Scan III	1,41	0,69	-0,72
Chan[78]	2016	CS	50	66,2	Victus	24	3	NA	Wallace	OPD-Scan III	1,35	0,67	-0,68
Zhang[79]	2022	CS	94	61,72	LenSX	3	2,4	Temporal	Donnenfeld Modified	Lenstar	1,36	0,7	-0,66
Wang[76]	2022	RCT	78	69	Catalys	1	2,4	Temporal	Paired 8 mm	IOL Master	0,97	0,34	-0,63
Chen[68]	2019	CS	60	64	Manual	3	NA	NA	NA	IOLMaster	1,1	0,51	-0,59
Lee[56]	2019	CS	14	NA	Catalys	1	2,2	NA	NA	Canon R-50	1,1	0,59	-0,51
Kwon[80]	2021	CS	27	69,4	Catalys	6	NA	NA	Julian Stevens v3	KR- 8800	1,44	0,98	-0,46
Ganesh[81]	2020	RCT	25	64,5	Catalys	6	2,8	Temporal	Donnenfeld	Pentacam	1,07	0,65	-0,42
Rani[82]	2020	CS	80	63	Catalys	3	NA	NA	Donnenfeld Modified	NA	0,85	0,47	-0,38
Löffler[83]	2017	CS	27	65	LenSX	3	NA	NA	Wang	Pentacam	0,97	0,63	-0,34
Stanojcic[84]	2021	RCT	51	69,8	Manual	12	2,4	NA	Donnenfeld	Pentacam	1,48	1,15	-0,33
Lopes[85]	2021	RCT	20	70,22	Catalys	3	NA	NA	Julian Stevens v3	IOLMaster	1,17	0,9	-0,27
Baharozian[86]	2017	CS	161	67	Catalys	1	NA	NA	Donnenfeld Modified	Pentacam	0,85	0,62	-0,23

CS: Case Series; RCT: Randomized clinical trial; NA: Not available; Diff: Difference between posteroperative (Post) and preoperative (Pre) magnitude of corneal astigmatism.

Unlike CCI and OCCI, for which few studies reported the percentage of eyes achieving different values of postoperative refractive astigmatism, up to 11 studies reported this information for AK incisions (Supplemental Table 1). It is of interest to note the study of Lee et al. [56] who evaluated the satisfaction in two groups implanted with diffractive MIOLs, one combined with AK and a control group without AK, reporting higher satisfaction rates in the AK group. Although the higher satisfaction of the AK group could not be explained by the application of the incisions, since postoperative corneal astigmatism was comparable in both groups (0.59 vs. 0.58 D), the higher preoperative corneal astigmatism of the AK group (1.10 vs. 0.51 D) suggests that the AK incisions were effective in patients implanted with MIOLs. A previous editorial by Porta[87] also suggested the effective correction of AK with the Lindstrom normogram reducing the mean preoperative astigmatism from 1.81 D to 0.56D in eyes implanted with the AMO Array SA40N MIOL.

### Limbal relaxing incisions

LRIs are a subset of AKs that are placed more peripherally than traditional arcuate keratotomy close to the limbus,[69] with a depth set at approximately 600 μm; therefore, they theoretically preserve higher optical quality of the cornea. [88]. In addition, they exhibit a lower frequency of postoperative glare, less discomfort, and a faster postoperative recovery of vision [88]. However, they correct a lesser amount of corneal astigmatism than incisions closer to the optical axis. [88]. Manual LRI has been shown to be both effective and safe. [41]. Again, LRIs may be performed with the use of FLS which has proven to be more reproducible [43].

Gills and Gayton’s LRI normogram[89] is based on the use of a 6.0 mm incision for 1.00 diopter of corneal astigmatism and pair incisions of 6.0 mm for 1.00 to 2.00 D of corneal astigmatism. Several authors have updated this nomogram with their own modifications either manually or with the use of FLS [90]. Likewise, Nichamin reported another LRI normogram [91, 92].

Donnenfeld’s LRI nomogram is based on the fact that for a corneal astigmatism of 0.5 D, it is necessary to make an incision of 45 degrees of arc, while for greater astigmatism two incisions are necessary, whose arcs vary depending on the magnitude of the previous astigmatism. It is important to note that in the same nomogram the author describes some exceptions where it is noted that for ATR astigmatism it is necessary to increase arc length by 5°. Similarly, for younger patients, it is necessary to increase arc length by 5°. On the contrary, for older patients, it is necessary to decrease arc length by 5°. Variations of this normogram have also been described in the literature, especially after the arrival of FSL technology. Table 4 shows the accuracy achieved through LRI, with only four studies (36%) showing a correction > 0.5D of corneal astigmatism. Only one study compared LRI with FSL versus the implantation of TIOLs, reporting no differences in the accuracy of astigmatism correction between groups [93].

Muftuoglu et al., demonstrated in a retrospective study that LRI can be an effective tool to reduce corneal astigmatism in patients implanted with MIOLs. In this study, the authors reported a decrease of astigmatism from 1.30 D to 0.59 D using a modified Gills and Gayton normogram [94]. This study was not included in Table 4 as the preoperative standard deviation of corneal astigmatism (0.65 D) exceeded the inclusion criteria. At 6-month 68% of eyes achieved a corneal astigmatism ≤ 0.50 D and 79% ≤ 1.00 D. An interesting finding in this study is that some patients required of Laser-Assisted In-Situ Keratomileusis (LASIK) retreatment, particularly in a group with mean preoperative astigmatism of 1.83 D.

In other study, Gangwani et al. compared the outcomes of toric MIOL in a group of 1.82D of corneal astigmatism and standard MIOL implantation combined with LRI in a group of 1.67 D. The astigmatism was reduced with both techniques but slightly more in the toric MIOL group with more predictable outcomes (0.45 vs. 0.72 D) [95].

### Intrastromal arcuate incisions (iAK)

As previously mentioned, with the advent of the FLS, the use of various types of corneal incisions can be performed using this approach, which has proven to be effective, safe, and especially reproducible when compared to the manual technique [96, 97]. An advancement of the FLS utilization technique is the ability to make intrastromal AK (iAK) type incisions without penetrating the Bowman layer or the Descemet membrane layer, which, unlike the classic transepithelial ones, do not open the incision. Thus, theoretically, they would not create an epithelial defect while maintaining its protective effect against corneal infections and decreasing postoperative pain. Moreover, the possibility of a persistent open corneal wound and epithelial ingrowth could be avoided [98]. According to the findings of this review, ten articles have reported their results regarding the use of iAK. Among the FLS devices used by the authors, the Catalys was used in seven articles, while the LensX has been used in two and the IntraLase in one. Rückl et al., [99] were the first to use this approach in 16 eyes. They observed a decrease of 0.87D with respect to corneal astigmatism. Regarding safety, the authors showed that all incisions were placed as intended without penetration in the Bowman or Descemet membrane. In a comparative retrospective study, Lopes[85] et al. compared the use of AK with FLS, using both a transepithelial AK and iAK in the same patient. The CI was 0.83 ± 0.71 and 0.68 ± 0.29 in the transepithelial group and the intrastromal group, respectively, showing no significant statistical difference. The percentage of eyes at ± 0.5D or less in postoperative corneal astigmatism was 30% in the transepithelial group and 40% in the intrastromal group. They discovered no serious postoperative complications in any group, although 20% of the patients in the transepithelial group reported discomfort. On the other hand, Ganesh et al[81] in a randomized clinical trial, demonstrated that although anterior penetrating and iAK incisions were effective in reducing preoperative astigmatism using the FLS, the transepithelial approach showed comparatively better correction. Recently, Wang et al. [76] in another large-sample randomized clinical trial demonstrated comparable outcomes.

The remainder of the studies report similar results regarding efficacy and safety. Consequently, according to the findings of this review, the use of iAK appears to be effective and safe in reducing corneal astigmatism in cases with low levels of preoperative corneal astigmatism. While most of the studies used the Catalys as the FLS, there appear to be no differences in the outcomes with the use of other FLS such as the IntraLase or the LensX. Despite the limited number of studies comparing the transepithelial vs. iAK technique, the former may be slightly more effective in reducing preoperative corneal astigmatism, resulting in more patients achieving ≤ 0.5 D in the postoperative period.

Table 5 displays the accuracy of iAK with 5 studies (representing 50%) resulting in a correction ≥ 0.5 D. According to this review, no studies have yet conducted a comparison between the accuracy of iAKs versus the implantation of TIOLs.

**Table 4:** Preoperative and postoperative magnitude of corneal astigmatism from keratometry reported in limbal relaxing incision (LRI) studies

Author	Year	Study Type	Eyes	Age_M	Technology	Follow (months)	Size	Location	Additional	Device	Pre	Post	Diff
Riaz[100]	2021	CS	118	72,21	Manual	12	2,4	NA	Donnenfeld	Pentacam	1,36	0,59	-0,77
Wang[101]	2016	CS	51	59	LenSX	3	2,2	NA	Donnenfeld	Orbscan II	1,41	0,65	-0,76
Freitas[102]	2014	RCT	32	71,75	Manual	6	2,75	Temporal	Donnenfeld	Orbscan II	1,32	0,74	-0,58
Eliwa[103]	2016	CS	32	61,07	Manual	12	2,2	Steepest	Donnenfeld	Keratron Scout	1,33	0,79	-0,54
Nanavaty[104]	2016	CS	80	76,71	Pentacam	3	2,4	Superior	Donnenfeld	Pentacam	1,42	0,99	-0,43
Blehm[105]	2021	RCT	38	69	LenSX	3	NA	NA	Woodcock	Lenstar	1,05	0,63	-0,42
Yoo[93]	2015	CS	23	53,63	IntraLase	5	2,2	Steeper	Donnenfeld	Manual	1,31	0,96	-0,35
Roberts[106]	2018	RCT	43	72,5	Manual	6	2,4	Middle Arcuate	Donnenfeld	IOL Master	1,5	1,17	-0,33
Blehm[105]	2021	RCT	38	69	Manual	3	NA	NA	Donnenfeld	Lenstar	0,98	0,7	-0,28
Muller-Jensen[107]	1999	CS	20	NA	Manual	6	4	NA	NA	Humphrey	0,87	0,81	-0,06
Lim[108]	2020	CS	154	71,44	Catalys	3	2,5	Temporal	Personal	IOL Master	0,87	0,85	-0,02

CS: Case Series; RCT: Randomized clinical trial; NA: Not available; Diff: Difference between postoperative (Post) and preoperative (Pre) magnitude of corneal astigmatism

**Table 5:** Preoperative and postoperative magnitude of corneal astigmatism from keratometry reported in intrastromal arcuate (iAK) studies

Author	Year	Study Type	Eyes	Age_M	Technology	Follow (months)	Size	Location	Additional	Device	Pre	Post	Diff
Rückl[99]	2013	CS	16	65	IntraLase	6	NA	NA	Paired	Keratron Scout	1,5	0,63	-0,87
Wang[76]	2022	RCT	125	68	Catalys	1	2,4	Temporal	Julian Stevens v3	IOL Master	0,97	0,39	-0,58
Lopes[85]	2021	RCT	20	70,22	Catalys	3	NA	NA	Julian Stevens v3	IOLMaster	1,22	0,66	-0,56
Byun[71]	2018	CS	89	63,8	Catalys	6	2,2	NA	Julian Stevens	Canon R50	1,16	0,63	-0,53
Stanojcic[84]	2021	RCT	53	70,1	LensX	12	2,4	NA	iFAKs	Pentacam	1,38	0,88	-0,5
Roberts[106]	2018	RCT	44	69,7	LensX	6	2,4	Middle Arcuate	Day	IOL Master	1,38	0,89	-0,49
Day[109]	2016	CS	186	62,1	Catalys	1	2,75	Temporal	Day	KR8100PA	1,21	0,74	-0,47
Day[110]	2016	CS	319	61,3	Catalys	1	2,75	Temporal	Day	KR8100PA	1,24	0,79	-0,45
Moon[111]	2021	CS	79	66,95	Catalys	1	2,2	NA	NA	Autokeratometer	1,23	0,8	-0,43
Ganesh[81]	2020	RCT	25	62	Catalys	6	2,8	Temporal	NA	Pentacam	1,23	0,9	-0,33

CS: Case Series; RCT: Randomized clinical trial; NA: Not available; Diff: Difference between postoperative (Post) and preoperative (Pre) magnitude of corneal astigmatism

### Complications of corneal incisions

In theory, one of the potential complications of CCI, especially in OCCI, may be the risk of endophthalmitis due to the additional penetrating incision compared to nonpenetrating techniques such as those in LRI or arcuate keratotomy. However, the risk of endophthalmitis in a standard cataract surgery with the use of antibiotic prophylaxis is indeed very low [112], [113]. Another potential disadvantage of OCCI may be that some surgeons might find it difficult to alter their preferred OCCI entry site for phacoemulsification [114]. Some complications have been reported, like early[115, 116] and late onset of microbial keratitis with FSL AK [117]. Likewise, keratitis complicated by endophthalmitis has also been reported several years ago [118]. Moreover a case report[119] and a series of corneal perforations were described during an arcuate keratotomy with FSL [120].

LRI induces low topographical irregularities, as well as minor glare and discomfort for the patient [70]. However, it is known that manual LRIs are surgeon dependent and result in some degree of variability and unpredictability [70]. Similarly, some complications have also been reported in the literature. In this regard, Moon et al. described a case of neurotrophic keratitis after performing cataract surgery together with LRI. In this case, the authors emphasize that the patient presented ectropion and lagophthalmos as risk factors, therefore they recommend to avoid this type of incisions in patients with a high risk of developing neurotrophic keratitis [121]. Similarly, Yu et al., reported a poor bilateral inferior LRI in a Graves ophthalmology patient [122]. Moreover, a devastating complication such as endophthalmitis has also been reported following LRI in combination with manual sutureless cataract extraction [123].

### Limitations

One of the drawbacks of conducting a cost-effectiveness study to meta-analyze the results of current published studies regarding corneal astigmatism correction with incisions is that there are few studies that report the percentage of patients who are in a certain range of residual refractive astigmatism. Most of them reported mean correction but not their ranges (Table 1), This fact is important because cost-effectiveness-based studies are determined not by the mean correction, but by the percentage of eyes reaching a certain value. For example, some studies may have the same mean, but the dispersion of the results is much greater in one group than in another. Therefore, we strongly recommend that the scientific community report not only the means but also the percentage of patients who reach a certain range of refractive results. In this particular case, the range of patients who present with different residual refractive astigmatism values. In addition, several normograms and their modifications of them by other authors have been described in the literature without reaching a definitive consensus on which is the best of them. Another limitation is that most of the included studies were retrospective case series studies. It is important to note that although clinical trials were included in this review, not all of them met the characteristics of randomized and blind clinical trials following the CONSORT guidelines. This is another important drawback that should be addressed in future studies. Likewise, it is worth mentioning that some articles used both eyes in their analyses. It is known that the use of data from both eyes could duplicate the information and therefore bias certain results since both eyes are normally correlated. [124]. Therefore, caution should be exercised when interpreting the results.

## Conclusion

Correction of low corneal astigmatism, between 0.50 D and 1.50 D, in patients operated on cataract surgery or refractive lens exchange is a topic of great interest for the anterior segment surgeon considering that this amount of astigmatism is presented in two-thirds of eyes submitted to cataract surgery [15]. In this review, we explored the current evidence regarding tolerance to astigmatism under-correction and its relationship with extension of the depth-of-focus. The decision criteria will depend on the objective of the patient and surgeon, either to maximize the far-distance vision or to extend the depth-of-focus with monofocal IOLs. Targeting a low myopic astigmatism with monofocal IOLs will slightly decrease far-distance vision, increasing the depth-of-focus. Thus, this clinical approach can be used to increase the spectacle independence in intermediate vision without implanting a presbyopia-correcting IOL.

However, this clinical approach is not transferable to eyes implanted with EDOF and MIOLs. In these cases, the tolerance of the uncorrected low astigmatism over UDVA will depend on the addition and optical design, as the EDOF lenses are more tolerant than diffractive MIOLs, particularly the small-aperture EDOF, which has shown the highest tolerance to uncorrected astigmatism. When there is a risk of decreasing UDVA due to preoperative corneal astigmatism, beyond the selection of a particular presbyopia IOL or the implantation of a TIOL, management of preoperative low corneal astigmatism (< 1.50 D) can be planned using several incision types. In this review, we have seen that the accuracy of correcting corneal astigmatism with CCI is at least questionable, with very few studies showing a decrease > 0.3 D. Furthermore, little evidence has been found for the use of OCCI. Conversely, the use of AK has shown the highest number of studies achieving a correction above 0.5 D, whereas less and comparable correction might be achieved with LRI and iAK.

## Electronic supplementary material

Below is the link to the electronic supplementary material.

Supplementary Material 1

Supplementary Material 2

## Data Availability

None.
