# Supplementary material for: Managing low corneal astigmatism in patients with presbyopia correcting intraocular lenses: a narrative review
Source: BMC Ophthalmol. 2023 Jun 6;23:254. doi: 10.1186/s12886-023-03003-2 (PMC10243013; doi:10.1186/s12886-023-03003-2)
Supplement: Supplementary file 1 — Supplementary Material 1 [file 12886_2023_3003_MOESM1_ESM.docx]

**Appending 1. Search strategy, inclusion criteria and data extraction**

A first reviewer covered the question of tolerance to astigmatism using the following keywords in a PubMed search (*astigmatism* *AND intraocular lenses* AND *multifocal*) combined with snowballing search techniques.

A second reviewer used a different search strategy focused in the research question of corneal incisions. The purpose was to identify all the available articles regarding the use of different corneal surgery techniques through incisions to reduce preoperative corneal astigmatism. For this purpose, the following search with Boolean operators was conducted in PubMed: *clear corneal incision AND astigmatism* finding a total of 354 articles. Other search strategy was *astigmatic keratotomy OR arcuate keratotomy AND cataract surgery* finding 279 articles; *peripheral corneal relaxing incisions,* finding 30 more references. Moreover, we also search using *limbal corneal relaxing incisions OR limbal relaxing incisions* finding 99 articles. After this first phase, all the available abstracts were analyzed. This first part of the review was started on 27 of January 2022. A new search was performed on 21 of August of 2022 following the same criteria with the same Boolean operators. For the comparison of corneal incisions with toric IOLs, studies coming from this search and including a toric IOL as comparator group were considered.

Inclusion criteria for corneal incision review question were studies which aim was to report the efficacy of the treatment of astigmatism through corneal incisions, reporting preoperative mean corneal astigmatism and postoperative corneal astigmatism. Only studies with mean preoperative corneal astigmatism between 0.625 D and 1.50 D with a standard deviation below 0.59 D were included. Among corneal incisions, we looked for studies that evaluated currently techniques described, among which stand out: clear corneal incision (CCI), limbal corneal incision, opposite clear corneal incision (OCCI), limbal relaxing incision (LRI) and arcuate keratectomy either manually or by means with the employment of FSL. Exclusion criteria were studies for which the type, location or size of the main incision was not described. Likewise, we excluded incision as frown or purely scleral incision, trapezoidal or transverse keratotomy. Similarly, studies where the objective was not to evaluate the efficacy of the treatment of astigmatism following a certain incision but the surgical induced astigmatism (SIA). We also rule out laser-based keratorefractive procedures and intraocular lens surgical procedures as piggyback to correct residual astigmatisms.

We only included articles written in English or Spanish, and discarded those written in other languages. Our objective was to identify case-based studies, both prospective and retrospective case series, and clinical trials based on cataract surgery or in virgin eyes therefore, we discarded those studies based on animals or experimentation or laboratory as well as reviews and those based on different surgeries than cataract as keratoplasty or glaucoma among other. Regarding data extraction, the main objective was to evaluate the efficacy of astigmatism treatment through corneal incisions. Thus, the mean preoperative and postoperative corneal astigmatism were selected as primary end-points of interest. The percentage of eyes achieving a particular value of postoperative corneal or refractive astigmatism were also extracted as secondary end-point together with study demographics: year, eyes, etc.
