# Supplementary material for: Managing low corneal astigmatism in patients with presbyopia correcting intraocular lenses: a narrative review
Source: BMC Ophthalmol. 2023 Jun 6;23:254. doi: 10.1186/s12886-023-03003-2 (PMC10243013; doi:10.1186/s12886-023-03003-2)
Supplement: Supplementary file 2 — Supplementary Material 2 [file 12886_2023_3003_MOESM2_ESM.docx]

| Supplementary Table 1. Percentage of eyes achieving a particular value of corneal astigmatism based on keratometry or postoperative refractive astigmatism | | | | | | | | | | | | | | | | | |
| --- | --- | --- | --- | --- | --- | --- | --- | --- | --- | --- | --- | --- | --- | --- | --- | --- | --- |
| **Author** | **Year** | **Eyes** | **Type** | **Technology** | **Follow (m)** | **CCI Size** | **CCI Location** | **Nomogram AK** | **Pre** | **K_0,25** | **K_0,50** | **K_0,75** | **K_1** | **Rx_0,25** | **Rx_0,50** | **Rx_0,75** | **Rx_1** |
| Ding[9] | 2022 | 33 | CCI | Manual | 3 | 2,4 | Temporal | NA | 1,28 | NA | NA | NA | NA | 47 | 75 | 89 | 100 |
| Ding[9] | 2022 | 32 | CCI | Manual | 3 | 3 | Steepest | NA | 1,15 | NA | NA | NA | NA | 34 | 56 | 72 | 91 |
| Chen[65] | 2020 | 138 | OCCI | FS LenSx | 1 | 2,8 | Steepest | NA | 1,31 | 5 | 40 | 69 | 86 | 9 | 54 | 75 | 89 |
| Chen[68] | 2019 | 60 | AK | Manual | 3 | NA | NA | NA (Verion guide) | 1,1 | NA | NA | NA | NA | 52 | 85 | 97 | NA |
| Chen[68] | 2019 | 60 | AK | Manual | 3 | NA | NA | NA | 1,1 | NA | NA | NA | NA | 38 | 70 | 87 | NA |
| Baharozian[86] | 2017 | 161 | AK | Catalys | 1 | NA | NA | Donnenfeld Modified | 0,85 | NA | 50 | NA | NA | NA | NA | NA | NA |
| Lopes[85] | 2021 | 20 | AK | Catalys | 3 | NA | NA | Julian Stevens v3 | 1,17 | NA | 30 | NA | 75 | NA | NA | NA | NA |
| Stanojcic[84] | 2021 | 51 | AK | Manual | 12 | 2,4 | Steepest | Donnenfeld | 1,48 | NA | NA | NA | NA | 15 | 35 | 56 | 72 |
| Wendelstein[12] | 2021 | 43 | AK | Victus | 3 | 2,2 | Temporal | Castrop | 1,45 | NA | NA | NA | NA | 60 | 97 | 99 | 100 |
| Rani[82] | 2020 | 80 | AK | Catalys | 3 | NA | NA | Donnenfeld Modified | 0,85 | NA | 69 | NA | 95 | NA | NA | NA | NA |
| Visco[75] | 2019 | 189 | AK | Lensar | 3 | NA | NA | Paired | 0,92 | NA | NA | NA | NA | 80 | 96 | 100 | 100 |
| Ganesh[81] | 2020 | 25 | AK | Catalys | 6 | 2,8 | Temporal | Donnenfeld | 1,07 | NA | NA | NA | NA | 56 | 80 | 92 | 100 |
| Wang[76] | 2022 | 78 | AK | Catalys | 1 | 2,4 | Temporal | Paired 8 mm | 0,97 | 1 | 10 | 19 | 58 | 63 | 78 | 91 | 95 |
| Wang[76] | 2022 | 45 | AK | Catalys | 1 | 2,4 | Temporal | Paired 9 mm | 1,04 | 2 | 18 | 24 | 49 | 58 | 93 | 98 | 100 |
| Eliwa[103] | 2016 | 32 | LRI | Manual | 12 | 2,2 | Steepest | Donnenfeld | 1,33 | NA | NA | NA | NA | 4 | 26 | 52 | 65 |
| Riaz[100] | 2021 | 118 | LRI | Manual | 12 | 2,4 | NA | Donnenfeld | 1,36 | NA | NA | NA | NA | 19 | 35 | 66 | 86 |
| Lim[108] | 2020 | 154 | LRI | Catalys | 3 | 2,5 | Temporal | Personal | 0,87 | NA | NA | NA | NA | 23 | 63 | 79 | 89 |
| Blehm[105] | 2021 | 38 | LRI | Manual | 3 | NA | NA | Donnenfeld | 0,98 | NA | NA | NA | NA | 79 | 89 | 95 | 97 |
| Blehm[105] | 2021 | 38 | LRI | LenSX | 3 | NA | NA | Woodcock | 1,05 | NA | NA | NA | NA | 79 | 95 | 97 | 97 |
| Wang[101] | 2016 | 51 | LRI | LenSX | 3 | 2,2 | NA | Donnenfeld | 1,41 | NA | NA | NA | NA | 33 | 50 | 67 | 87 |
| Roberts[106] | 2018 | 43 | LRI | Manual | 6 | 2,4 | Middle Arcuate | Donnenfeld | 1,5 | NA | NA | NA | NA | 7 | 19 | 64 | 74 |
| Lopes[85] | 2021 | 20 | iAK | Catalys | 3 | NA | NA | Julian Stevens v3 | 1,22 | NA | 40 | NA | 90 | NA | NA | NA | NA |
| Stanojcic[84] | 2021 | 53 | iAK | LensX | 12 | 2,4 | NA | iFAKs | 1,38 | NA | NA | NA | NA | 19 | 44 | 65 | 77 |
| Day[109] | 2016 | 186 | iAK | Catalys | 1 | 2,8 | Temporal | Day | 1,21 | 3 | 20 | 47 | 74 | NA | NA | NA | NA |
| Roberts[106] | 2018 | 44 | iAK | LensX | 6 | 2,4 | Middle Arcuate | Day | 1,38 | NA | NA | NA | NA | 2 | 42 | 56 | 75 |
| Ganesh[81] | 2020 | 25 | iAK | Catalys | 6 | 2,8 | Temporal | NA | 1,23 | NA | NA | NA | NA | 4 | 76 | 92 | 100 |
| Wang[76] | 2022 | 125 | iAK | Catalys | 1 | 2,4 | Temporal | Julian Stevens v3 | 0,97 | 4 | 14 | 34 | 58 | 55 | 76 | 90 | 95 |

K_: Anterior Surface Corneal Astigmatism; Rx_: Refractive astigmatism; iAK: Intrastromal arcuate; AK: Arcuate; LRI: Limbal relaxing; OCCI: Opposite clear corneal; CCI: Clear corneal
